# *In vitro* activity of the siderophore cephalosporin, cefiderocol, against molecularly characterized, carbapenem-non-susceptible Gram-negative bacteria from Europe

**DOI:** 10.1093/jacamr/dlaa060

**Published:** 2020-08-25

**Authors:** Christopher Longshaw, Davide Manissero, Masakatsu Tsuji, Roger Echols, Yoshinori Yamano

**Affiliations:** 1 Infectious Diseases, Shionogi B.V., London, UK; 2 Marketed Product Regulatory Affairs, Shionogi & Co., Ltd., Osaka, Japan; 3 ID3C, LLC, Easton, CT, USA; 4 Pharmaceutical Research Division, Shionogi & Co., Ltd., Osaka, Japan

## Abstract

**Objectives:**

Many carbapenem-resistant (CR) Gram-negative (GN) pathogens exhibit MDR, meaning few therapeutic options are available for CR-GN infections. Cefiderocol, a siderophore cephalosporin, has demonstrated *in vitro* efficacy against CR-GN bacteria. In the SIDERO-CR-2014–2016 surveillance study, European clinical isolates comprising carbapenem-non-susceptible (CarbNS) Enterobacterales and MDR non-fermenters were tested against cefiderocol and comparators.

**Methods:**

Cefiderocol MICs were determined using iron-depleted CAMHB, and comparators using CAMHB, per recommended CLSI methodology. Carbapenemase gene profiles were determined using PCR.

**Results:**

Isolates (*N = *870) from 23 European countries comprised CarbNS Enterobacterales (*n = *457), MDR *Pseudomonas aeruginosa* (*n = *177) and MDR *Acinetobacter baumannii* (*n = *236). The most common carbapenemases were KPC (52%), OXA-48-like (19%), VIM (14%) and NDM (8%) in Enterobacterales, VIM (41%) in *P. aeruginosa* and OXA-23-like (57%) and OXA-24/40-like (37%) in *A. baumannii*. Most carbapenemase-producing isolates (65%) co-carried ESBLs. Approximately half of *P. aeruginosa* isolates were negative for carbapenemases, compared with 10% of Enterobacterales and 3% of *A. baumannii*. A similar proportion of Enterobacterales were susceptible to cefiderocol (81.6%; 79.0% of VIM producers; 51.4% of NDM producers; based on EUCAST breakpoint values) compared with comparator antimicrobial agents, including colistin (76.4%; 93.5% of VIM producers; 78.4% of NDM producers) and ceftazidime/avibactam (76.6%; 1.6% of VIM producers; 2.7% of NDM producers). Of *P. aeruginosa* isolates, 98.3% were susceptible to cefiderocol (100% of VIM producers), similar to colistin (100%). Against *A. baumannii*, 94.9% had cefiderocol MIC ≤2 mg/L and 93.6% of isolates were susceptible to colistin.

**Conclusions:**

Cefiderocol demonstrated potent activity against CarbNS and MDR GN bacteria, including non-fermenters and a wide variety of MBL- and serine-β-lactamase-producing strains.

## Introduction

Carbapenems are a treatment of choice for many infections caused by Gram-negative (GN) bacteria. Globally, the incidence of carbapenem-resistant (CR) GN bacteria is increasing,^1^^,^^2^ with resistance largely due to the production of β-lactamase enzymes.^3^ There are very few therapeutic options for CR-GN bacterial infections, as many CR pathogens exhibit MDR, including resistance to alternative β-lactams (e.g. cephalosporins and penicillins), as well as other common drug classes such as aminoglycosides and fluoroquinolones.^1^^,^^3^ At the time of treatment initiation, and even after pathogen identification, molecular characterization of the mechanism of carbapenem resistance is not available and the identity of a specific β-lactamase conferring resistance is likely to be unknown. These unknowns can lead to a delay in the time taken to begin appropriate antibiotic therapy, which can negatively impact the length of hospital stay, in-hospital costs and mortality.^4^

Cefiderocol is a novel siderophore cephalosporin, which was developed for the treatment of MDR GN bacteria, including those resistant to carbapenems. Cefiderocol has recently been approved in Europe for the treatment of infections due to aerobic GN organisms in adults with limited treatment options^5^ and in the USA for the treatment of complicated urinary tract infections (cUTIs), including pyelonephritis, caused by susceptible GN organisms in adult patients with limited treatment options.^6^

The structure of cefiderocol is based around a cephalosporin backbone with the addition of a catechol moiety at the 3-position side chain. The cephalosporin core enables cefiderocol to act like other cephalosporins, binding primarily to PBPs and killing bacterial cells by inhibition of peptidoglycan cell wall biosynthesis. Cefiderocol differs from other cephalosporins in that the catechol moiety chelates ferric (Fe-III) iron, mimicking natural siderophores, allowing cefiderocol to exploit the bacteria’s own active receptor-mediated iron transport system to cross the outer membrane.^7^^,^^8^ The resulting increase in periplasmic concentration circumvents non-specific resistance due to porin loss or efflux and enhances cefiderocol’s activity relative to carbapenems, other cephalosporins and β-lactam/β-lactamase inhibitor combinations.^6^^,^^9^^,^^10^ Cefiderocol is active against CR-GN bacteria harbouring β-lactamases from all Ambler classes, including KPC, VIM, IMP, NDM and OXA carbapenemases, and is active against ESBL-producing *Escherichia coli* and *Klebsiella pneumoniae*, as well as meropenem-resistant *Pseudomonas aeruginosa* and *Acinetobacter baumannii.*^11–13^

Susceptibility testing of cefiderocol by broth microdilution requires an iron-depleted medium to promote the natural production of siderophores by bacterial cells. Iron-depleted *in vitro* conditions are essential in order to mimic the hypoferremic conditions encountered by bacteria in the human body during infection.^7^^,^^11^^,^^14^ Determination of cefiderocol MICs using iron-depleted (iron concentration <0.03 mg/L) CAMHB (ID-CAMHB)^15^ has been shown to give reproducible results that correlate with *in vivo* efficacy^16^ and ID-CAMHB has been recommended as the standard medium for cefiderocol MIC determination by CLSI.^17^

In the SIDERO-CR surveillance study, CR and MDR clinical isolates of GN bacteria collected from patients between 2014 and 2016 were tested against cefiderocol and comparators using recommended CLSI broth microdilution methodology.^18^ In this report, we focus on the clinical isolates provided for the SIDERO-CR study by European hospitals.

## Methods

Full methodology for the SIDERO-CR study and molecular characterization using PCR has been published previously (see Supplementary materials, available as Supplementary data at *JAC-AMR* Online).^19^^,^^20^ The SIDERO-CR isolates were taken from a 2014–16 International Health Management Associates, Inc. (IHMA; Schaumburg, IL, USA) surveillance collection based on their known antimicrobial susceptibility phenotypes and/or their species identification.^19^ Isolates were screened for the presence of clinically relevant β-lactamase genes, including Ambler class A ESBLs (e.g. SHV, CTX-M) and carbapenemases (e.g. KPC, GES), class B MBLs (e.g. NDM, VIM), class C plasmid-mediated AmpC-type β-lactamases and class D β-lactamases (e.g. OXA-type).^20^ SIDERO-CR-2014–2016 included isolates of GN bacilli from 23 European countries [Austria (3 sites), Belgium (5), Croatia (2), Czech Republic (4), Denmark (1), France (5), Germany (8), Greece (4), Hungary (3), Ireland (1), Italy (18), Latvia (1), Lithuania (2), the Netherlands (1), Poland (3), Portugal (6), Romania (5), Russia (10), Serbia (2), Slovenia (1), Spain (15), Turkey (6) and the UK (3)].

Included in the SIDERO-CR-2014–2016 European test set were carbapenem-non-susceptible (CarbNS) phenotypes of Enterobacterales isolates (*n = *457), defined as having a meropenem MIC of ≥2 mg/L. Isolates of *P. aeruginosa* (*n = *177) and *A. baumannii* (*n = *236) were included if they demonstrated an amikacin-resistant (MIC ≥32 mg/L), ciprofloxacin-resistant (MIC ≥4 mg/L) and imipenem-resistant (MIC ≥16 mg/L) MDR phenotype.

MICs were determined for cefiderocol, cefepime, ceftazidime/avibactam, ceftolozane/tazobactam, ciprofloxacin, colistin and meropenem by broth microdilution according to CLSI guidelines.^18^ Susceptibilities of all antibiotics, with the exception of cefepime when tested against *A. baumannii* (CLSI breakpoint ≤8 mg/L),^18^ were interpreted using EUCAST breakpoints.^5^^,^^21^ Enterobacterales and *P. aeruginosa* are considered susceptible to cefiderocol at MIC ≤2 mg/L (resistant >2 mg/L).^5^ Cefiderocol was tested using ID-CAMHB, while all other antimicrobial agents were tested using standard CAMHB. Quality control testing was performed on each day of testing.

### Ethics

Ethics approval was not required as all *in vitro* samples were anonymized.

## Results

### Bacterial isolates

The majority of SIDERO-CR-2014–2016 European isolates (*N = *870) were from Italy [217 (24.9%)], Greece [128 (14.7%)] and Russia [91 (10.5%)].

Respiratory tract infections (RTIs) were the most common isolate source [385 (44.3%)]; the majority were non-fermenters [241/385 (62.6%)], consisting of *A. baumannii* [135/385 (35.1%)] and *P. aeruginosa* (106/385 [27.5%]). *K. pneumoniae* (120/385 [31.2%]) was the most common Enterobacterales species in RTIs (Table S1). Urinary tract infections (UTIs) were the next most common source (*n = *157; 18.0%), followed by intra-abdominal infections (IAIs; *n = *125; 14.4%), surgical site infections (SSIs; *n = *89; 10.2%) and bloodstream infections (BSIs; *n = *85; 9.8%). *K. pneumoniae* was the most common pathogen in UTIs [59/157 (37.6%)], SSIs [57/89 (64.0%)] and BSIs [47/85 (55.3%)] while *A. baumannii* was the most common in IAIs [46/125 (36.8%)].

### In vitro activity by species

Cefiderocol exhibited *in vitro* activity against a variety of CarbNS-GN bacteria in SIDERO-CR-2014–2016 European isolates. Overall, 772/870 (88.7%) had a cefiderocol MIC of ≤2 mg/L; 547/634 (86.3%) of Enterobacterales and *P. aeruginosa* isolates were susceptible to cefiderocol and 224/236 (94.9%) of *A. baumannii* isolates had a cefiderocol MIC of ≤2 mg/L.

Of CarbNS Enterobacterales, 81.6% were cefiderocol susceptible (MIC_90_ = 4 mg/L) (Table 1). The proportion of all Enterobacterales isolates susceptible to comparators was similar to colistin (76.4% susceptible) and ceftazidime/avibactam (76.6% susceptible). Of *K. pneumoniae* isolates (*n = *332), 82.8% were susceptible to cefiderocol, while 71.7% were susceptible to colistin and 88.9% to ceftazidime/avibactam.

**Table 1.** *In vitro* activity of cefiderocol and comparators against CarbNS- and MDR-GN bacteria with ≥10 European isolates (SIDERO-CR-2014–2016)

Species (*n*)	Antibacterial	MIC (mg/L)			MIC interpretation^a^
		range	MIC_50_	MIC_90_	% susceptible
Enterobacterales (457)					
	cefiderocol	0.015–32	1	4	81.6
	cefepime	0.12 to >64	>64	>64	0.9
	ceftazidime/avibactam	≤0.06 to >64	2	>64	76.6
	ceftolozane/tazobactam	0.25 to >64	>64	>64	0.4
	ciprofloxacin	≤0.12 to >8	>8	>8	4.2
	colistin	≤0.25 to >8	0.5	>8	76.4
	meropenem	2 to >64	16	>64	4.2
*E. coli* (24)					
	cefiderocol	0.12–4	0.5	4	87.5
	cefepime	8 to >64	64	>64	0
	ceftazidime/avibactam	0.12 to >64	0.5	>64	79.2
	ceftolozane/tazobactam	16 to >64	64	>64	0
	ciprofloxacin	≤0.12 to >8	>8	>8	8.3
	colistin	≤0.25–1	0.5	0.5	100
	meropenem	2 to >64	8	16	16.7
*K. pneumoniae* (332)					
	cefiderocol	0.03–32	1	4	82.8
	cefepime	0.5 to >64	>64	>64	0.3
	ceftazidime/avibactam	0.12 to >64	2	64	88.9
	ceftolozane/tazobactam	8 to >64	>64	>64	0
	ciprofloxacin	≤0.12 to >8	>8	>8	1.8
	colistin	≤0.25 to >8	0.5	>8	71.7
	meropenem	2 to >64	32	>64	7.8
*Serratia marcescens* (10)					
	cefiderocol	0.25–4	2	4	70.0
	cefepime	64 to >64	>64	>64	0
	ceftazidime/avibactam	1 to >64	>64	>64	40.0
	ceftolozane/tazobactam	64 to >64	>64	>64	0
	ciprofloxacin	1 to >8	8	>8	0
	colistin	>8 to >8	>8	>8	0
	meropenem	8 to >64	>64	>64	0
*Citrobacter freundii* (11)					
	cefiderocol	0.015–8	0.5	2	90.9
	cefepime	1 to >64	32	>64	9.1
	ceftazidime/avibactam	≤0.06 to >64	1	>64	72.7
	ceftolozane/tazobactam	4 to >64	64	>64	0
	ciprofloxacin	0.25 to >8	>8	>8	9.1
	colistin	0.5–1	0.5	1	100
	meropenem	2–16	4	8	18.2
*Klebsiella oxytoca* (13)					
	cefiderocol	0.06–4	0.5	2	92.3
	cefepime	4 to <64	32	>64	0
	ceftazidime/avibactam	0.5 to >64	1	>64	53.8
	ceftolozane/tazobactam	4 to >64	32	>64	0
	ciprofloxacin	≤0.12 to >8	>8	>8	15.4
	colistin	≤0.25 to >8	0.5	1	92.3
	meropenem	2–64	8	32	7.7
*E. cloacae* (59)					
	cefiderocol	0.25–32	2	4	69.5
	cefepime	2 to >64	64	>64	0
	ceftazidime/avibactam	0.25 to >64	>64	>64	18.6
	ceftolozane/tazobactam	1 to >64	>64	>64	1.7
	ciprofloxacin	≤0.12 to >8	>8	>8	5.1
	colistin	≤0.25–8	0.5	2	98.3
	meropenem	2 to >64	8	>64	5.1
*P. aeruginosa* (177)					
	cefiderocol	0.004–8	0.25	1	98.3
	cefepime	1 to >64	32	>64	13.6
	ceftazidime/avibactam	1 to >64	32	>64	34.5
	ceftolozane/tazobactam	0.5 to >64	>64	>64	22.6
	ciprofloxacin	1 to >8	>8	>8	0
	colistin	≤0.25–2	1	1	100
	meropenem	1 to >64	32	>64	2.3
*A. baumannii* (236)					
	cefiderocol	0.015 to >64	0.12	1	NA
	cefepime^b^	4 to >64	64	>64	4.7
	ceftazidime/avibactam	≤0.06 to >64	32	>64	NA
	ceftolozane/tazobactam	1 to >64	16	>64	NA
	ciprofloxacin	>8 to >8	>8	>8	0
	colistin	≤0.25 to >8	0.5	1	93.6
	meropenem	1 to >64	64	>64	0.8

Colistin MIC measurement range was 0.25–8 mg/L. Ciprofloxacin MIC measurement range was 0.12–8 mg/L.

MIC_n_, MIC for *n*% of isolates tested; NA, not applicable (no breakpoint available).

a Based on EUCAST breakpoints.^5^^,^^21^

b Based on CLSI breakpoint.^17^

The cefiderocol MIC_90_ was 1 mg/L against MDR *P. aeruginosa* and 98.3% of isolates were cefiderocol susceptible (MIC ≤2 mg/L). Colistin demonstrated a similar level of activity (100% susceptible) to cefiderocol, while other comparators were active against <35% of isolates.

Against MDR *A. baumannii*, the cefiderocol MIC_90_ was 1 mg/L and 94.9% of isolates had a cefiderocol MIC of ≤2 mg/L. Of the comparators, only colistin demonstrated activity (93.6% susceptible). The MIC ranges for cefiderocol were 0.004 − 8 mg/L for *P. aeruginosa* and 0.03 − 32 mg/L for *K. pneumoniae*, while the range against *A. baumannii* was somewhat wider (0.015 to >64 mg/L).

In total, the proportion of Enterobacterales and *P. aeruginosa* isolates non-susceptible to cefiderocol (MIC >2 mg/L) was 13.7% (87/634); these isolates consisted mainly of CarbNS *K. pneumoniae* (*n = *57) and *Enterobacter cloacae* (*n = *18) (Figure S1). There were also 12 *A. baumannii* isolates with a cefiderocol MIC of >2 mg/L [from Russia (*n = *7), Italy (*n = *2), Denmark (*n = *1), Portugal (*n = *1) and Turkey (*n = *1)].

**Figure 1.**
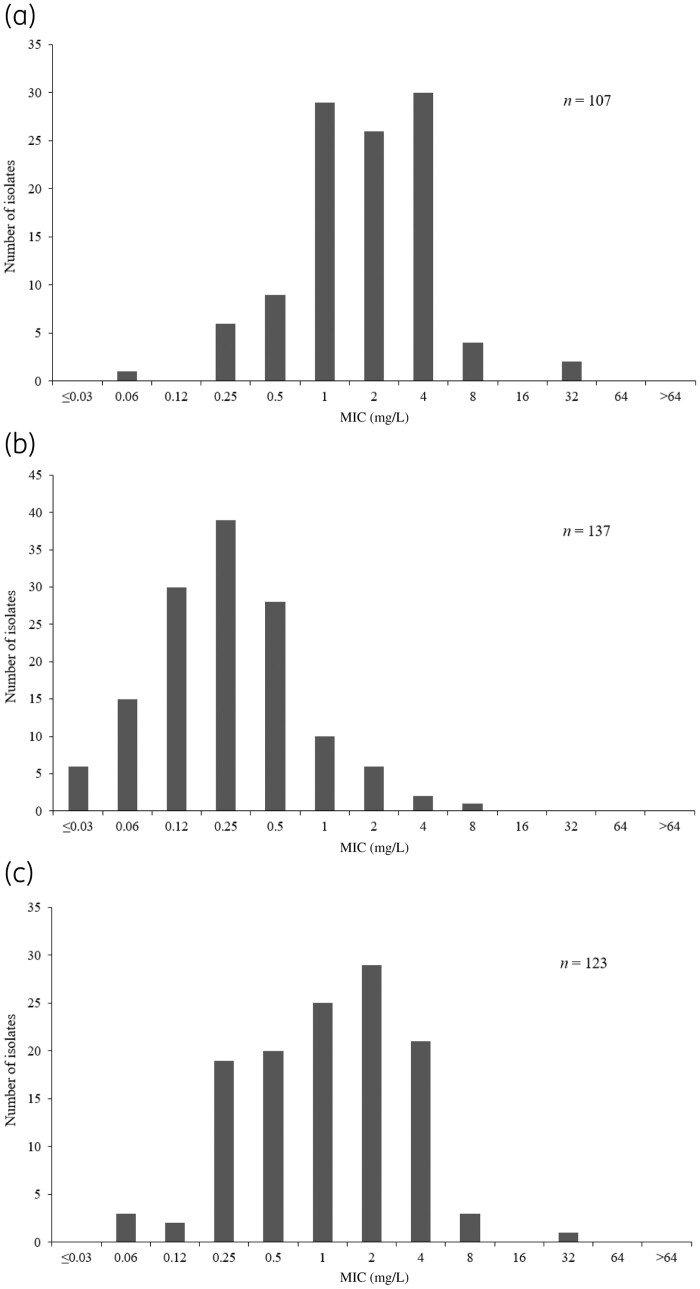
Cefiderocol MIC distribution against (a) ceftazidime/avibactam-resistant Enterobacterales, (b) ceftolozane/tazobactam-resistant *P. aeruginosa* and (c) colistin-resistant European SIDERO-CR-2014–2016 isolates. Based on EUCAST breakpoints for susceptibility (ceftazidime/avibactam: 8 mg/L; ceftolozane/tazobactam: 4 mg/L; colistin: 2 mg/L).^21^

Notably, 66.4% of ceftazidime/avibactam-resistant Enterobacterales remained susceptible to cefiderocol (*n = *107; cefiderocol MIC_90_* = *4 mg/L; range: 0.06 − 32 mg/L), as did 97.8% of ceftolozane/tazobactam-resistant *P. aeruginosa* (*n = *137; cefiderocol MIC_90_* = *1 mg/L; range: ≤0.03 − 8 mg/L) and all colistin-resistant isolates (*n = *123; cefiderocol MIC_90_* = *4 mg/L; range: 0.06 − 32 mg/L) (Figure 1). Of colistin-resistant Enterobacterales and *P. aeruginosa* isolates (*n = *108), 76.9% were cefiderocol susceptible; all 15 colistin-resistant *A. baumannii* isolates had cefiderocol MICs of ≤2 mg/L.

### Carbapenemase profiles of European SIDERO-CR-2014–2016 isolates

**Figure 2.**
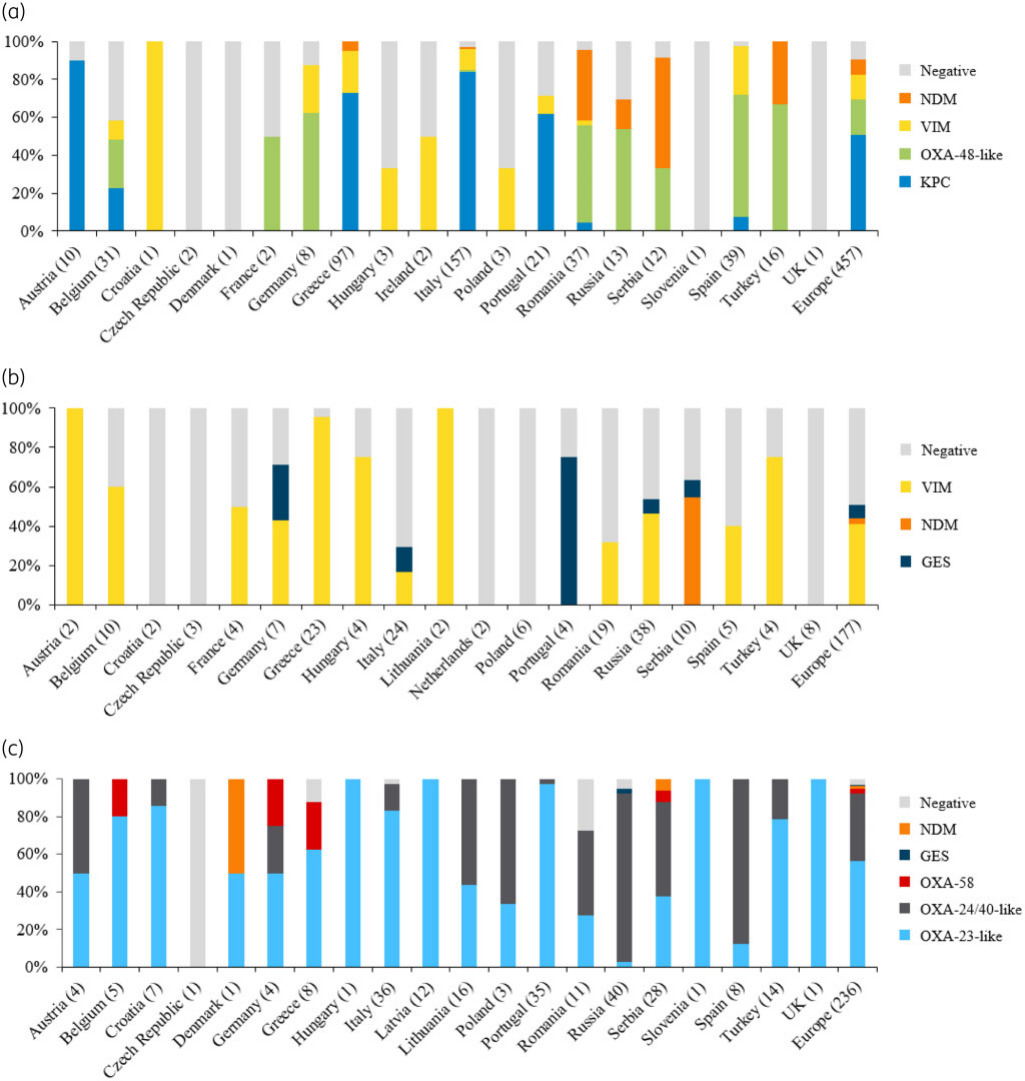
Carbapenemases identified in European SIDERO-CR-2014–2016 isolates, by country, for (a) CarbNS Enterobacterales, (b) MDR *P. aeruginosa* and (c) MDR *A. baumannii*. (a) Includes two isolates co-carrying KPC and VIM carbapenemases (Greece) and eight isolates co-carrying OXA-48 and NDM carbapenemases (six in Romania and two in Turkey). (b) Includes one isolate co-carrying GES and VIM carbapenemases (Russia) and one isolate co-carrying GES and NDM carbapenemases (Serbia). (c) Includes two isolates co-carrying OXA-24/40-like, OXA-58 and NDM carbapenemases (Serbia) and one isolate co-carrying OXA-23-like and NDM carbapenemases (Denmark).

In total, 28 subclasses of carbapenemase were identified, along with 37 ESBL (e.g. CTX-M) and older-spectrum β-lactamase (OSBL; e.g. SHV and TEM) subclasses,^22^ representing all Ambler classes. Key carbapenemases by country are shown in Figure 2. Across all isolates, the most common carbapenemase produced by Enterobacterales was KPC (52.1%), followed by OXA-48-like (18.6%), VIM (13.6%) and NDM (8.1%). Among *P. aeruginosa* isolates, VIM (41.2%) was the most common carbapenemase. OXA-23-like (57.2%) and OXA-24/40-like (37.3%) producers accounted for the majority of *A. baumannii* isolates. Most isolates produced multiple β-lactamases, e.g. 34/37 (91.9%) NDM-1 Enterobacterales also harboured ESBLs and OSBLs. A proportion of all isolates, phenotypically non-susceptible to meropenem, were negative for carbapenemase genes, with a greater proportion of *P. aeruginosa* (49.7%) being carbapenemase negative compared with Enterobacterales (9.8%) and *A. baumannii* (3.4%).

Italy (*n = *157) and Greece (*n = *97) provided the most CarbNS Enterobacterales isolates. Most of these isolates produced KPC (Italy: 84.1%; Greece: 74.2%) or VIM (Italy: 11.5%; Greece: 22.7%); however, NDM was less prevalent in Italy (0.6%) than in Greece (5.2%). OXA-48-like carbapenemases (including OXA-48, -162, -181, -232, -244 and -505^23^^,^^24^) were most common in Turkey [12/16 (75.0%)], Spain [25/39 (64.1%)] and Germany [5/8 (62.5%)], while NDM was most common in Serbia [7/12 (58.3%)] and was prevalent in Romania [16/37 (43.2%)].

In countries providing ≥8 isolates, the majority of carbapenemase-producing MDR *P. aeruginosa* were VIM producers, with high incidence in Greece [22/23 (95.7%)]. NDM-producing *P. aeruginosa* isolates were only apparent in Serbia, representing 6/10 samples.

Analysis of MDR *A. baumannii* isolates from countries with ≥8 available isolates established that the majority produced OXA-23-like carbapenemases,^23^ which were prevalent in Portugal [34/35 (97.1%)], Italy [30/36 (83.3%)] and Turkey [11/14 (78.6%)], while OXA-24/40-like carbapenemases (including OXA-24/40 and -72^22^) were the most common in Russia [36/40 (90.0%)]. The distribution of key carbapenemase subclasses by country and pathogen is displayed in Figure S2.

### In vitro activity by carbapenemase

Cefiderocol MIC_90_ values were ≤4 mg/L for all carbapenemases across all strains where ≥10 isolates were available for testing (Table 2). Across all isolates, cefiderocol MIC_90_ values were ≤4 mg/L for both VIM (total across all species: *n = *135) and NDM (total across all species: *n = *46) MBL producers. Isolates producing OXA-48-like (*n = *85), OXA-23-like (*n = *135) and OXA-24/40-like (*n = *88) carbapenemases had cefiderocol MIC_90_ values of ≤4 mg/L.

**Table 2.** Cefiderocol MIC distribution by carbapenemase for European isolates (SIDERO-CR-2014–2016)

Carbapenemase (*n*)	Number of isolates at cefiderocol MIC (mg/L)													MIC_50_ (mg/L)	MIC_90_ (mg/L)	MIC ≤2 mg/L (%)
	≤0.03	0.06	0.12	0.25	0.5	1	2	4	8	16	32	64	≥64			
KPC (238)	2	6	11	24	35	70	51	35	4	0	0	0	0	1	4	83.6
GES (13)	0	4	3	4	2	0	0	0	0	0	0	0	0	0.12	0.25	100
VIM (135)	5	13	24	27	17	19	17	11	2	0	0	0	0	0.25	2	90.4
NDM (46)	0	0	0	3	3	8	13	16	2	0	1	0	0	2	4	58.7
OXA-23-like (135)	19	40	29	22	12	7	1	3	2	0	0	0	0	0.12	0.5	96.3
OXA-24/40-like (88)	1	19	23	22	11	2	4	0	1	2	0	1	2	0.25	2	93.2
OXA-48-like (85)	4	3	0	17	16	23	12	10	0	0	0	0	0	1	4	88.2
OXA-58 (6)	0	0	1	2	1	2	0	0	0	0	0	0	0	NA	NA	100
No carbapenemase (142)	10	6	12	33	26	27	16	8	2	0	1	0	1	0.5	2	91.5
No β-lactamase (79)	9	5	11	23	11	16	2	2	0	0	0	0	0	0.25	1	97.5

Includes two isolates co-carrying KPC and VIM carbapenemases, eight isolates co-carrying OXA-48 and NDM carbapenemases, one isolate co-carrying GES and VIM carbapenemases, one isolate co-carrying GES and NDM carbapenemases, two isolates co-carrying OXA-24/40-like, OXA-58 and NDM carbapenemases and one isolate co-carrying OXA-23-like and NDM carbapenemases.

MIC_n_, minimum concentration inhibiting *n*% of isolates tested; NA, not applicable (<10 isolates).

The activity of cefiderocol and comparators by carbapenemase is summarized in Table 3. The cefiderocol MIC_90_ of KPC-producing isolates was 4 mg/L, with 83.6% being cefiderocol susceptible at the EUCAST breakpoint of ≤2 mg/L, but with 98.3% being cefiderocol susceptible at the CLSI breakpoint of ≤4 mg/L. Ceftazidime/avibactam demonstrated potent activity against 97.9% of KPC-producing Enterobacterales by both EUCAST and CLSI breakpoints. Similarly, against OXA-48-like-producing Enterobacterales, only cefiderocol and ceftazidime/avibactam (both 88.2% susceptible) demonstrated efficacy in >75% isolates. Against VIM-producing Enterobacterales, only cefiderocol (79.0% susceptible) and colistin (93.5% susceptible) demonstrated notable activity. The proportion of susceptible NDM-producing Enterobacterales isolates was higher for cefiderocol (51.4%) and colistin (78.4%) versus all other comparators (<3%).

**Table 3.** *In vitro* activity of cefiderocol and comparators against carbapenemases produced by CarbNS- and MDR-GN bacteria in European isolates (SIDERO-CR-2014–2016)

Species/carbapenemase (*n*)	Antibacterial	MIC (mg/L)			MIC interpretation^a^
		range	MIC_50_	MIC_90_	% susceptible
Enterobacterales^b^					
KPC (238)					
	cefiderocol	0.03–8	1	4	83.6
	cefepime	0.5 to >64	>64	>64	0.4
	ceftazidime/avibactam	0.12 to >64	1	8	97.9
	ceftolozane/tazobactam	4 to >64	>64	>64	0
	ciprofloxacin	≤0.12 to >8	>8	>8	2.9
	colistin	≤0.25 to >8	0.5	>8	73.9
	meropenem	2 to >64	32	>64	1.3
OXA-48-like (85)					
	cefiderocol	0.015–4	1	4	88.2
	cefepime	1 to >64	>64	>64	1.2
	ceftazidime/avibactam	0.25 to >64	1	>64	88.2
	ceftolozane/tazobactam	4 to >64	>64	>64	0
	ciprofloxacin	≤0.12 to >8	>8	>8	1.2
	colistin	≤0.25 to >8	0.5	>8	67.1
	meropenem	2 to >64	8	>64	12.9
VIM (62)					
	cefiderocol	0.06–8	1	4	79.0
	cefepime	1 to >64	>64	>64	1.6
	ceftazidime/avibactam	≤0.06 to >64	>64	>64	1.6
	ceftolozane/tazobactam	32 to >64	>64	>64	0
	ciprofloxacin	0.25 to >8	>8	>8	6.5
	colistin	≤0.25 to >8	0.5	1	93.5
	meropenem	2 to >64	16	>64	4.8
NDM (37)					
	cefiderocol	1–32	2	4	51.4
	cefepime	16 to >64	>64	>64	0
	ceftazidime/avibactam	4 to >64	>64	>64	2.7
	ceftolozane/tazobactam	64 to >64	>64	>64	0
	ciprofloxacin	2 to >8	>8	>8	0
	colistin	≤0.25 to >8	0.5	>8	78.4
	meropenem	4 to >64	64	>64	0
*P. aeruginosa* ^c^					
VIM (73)					
	cefiderocol	0.015–2	0.12	0.5	100
	cefepime	2 to >64	32	>64	4.1
	ceftazidime/avibactam	4 to >64	32	>64	6.8
	ceftolozane/tazobactam	32 to >64	>64	>64	0
	ciprofloxacin	2 to >8	>8	>8	0
	colistin	≤0.25–2	1	1	100
	meropenem	2 to >64	64	>64	1.4
GES (12)					
	cefiderocol	0.06–0.5	0.12	0.25	100
	cefepime	8 to >64	32	64	33.3
	ceftazidime/avibactam	2 to >64	4	32	75.0
	ceftolozane/tazobactam	8 to >64	32	>64	0
	ciprofloxacin	8 to >8	>8	>8	0
	colistin	0.5–2	1	1	100
	meropenem	8 to >64	64	>64	0
NDM (6)					
	cefiderocol	0.25–0.5	NA	NA	100
	cefepime	64 to >64	NA	NA	0
	ceftazidime/avibactam	>64 to >64	NA	NA	0
	ceftolozane/tazobactam	>64 to >64	NA	NA	0
	ciprofloxacin	>8 to >8	NA	NA	0
	colistin	1–1	NA	NA	100
	meropenem	64 to >64	NA	NA	0
non-carbapenemase-producing (88)					
	cefiderocol	0.004–8	0.25	1	96.6
	cefepime	1 to >64	32	>64	19.3
	ceftazidime/avibactam	1 to >64	8	64	53.4
	ceftolozane/tazobactam	0.5 to >64	16	>64	45.5
	ciprofloxacin	1 to >8	>8	>8	0
	colistin	≤0.25–2	1	2	100
	meropenem	1 to >64	8	32	3.4
*A. baumannii* ^d^					
OXA-23-like (135)					
	cefiderocol	0.015–8	0.25	0.5	NA
	cefepime^e^	8 to >64	64	>64	3.7
	ceftazidime/avibactam	4 to >64	64	>64	NA
	ceftolozane/tazobactam	4 to >64	32	>64	NA
	ciprofloxacin	>8 to >8	>8	>8	0
	colistin	≤0.25 to >8	0.5	1	94.8
	meropenem	8 to >64	64	>64	0
OXA-24/40-like (88)					
	cefiderocol	0.03 to >64	0.25	2	NA
	cefepime^e^	8 to >64	64	>64	2.3
	ceftazidime/avibactam	4 to >64	32	64	NA
	ceftolozane/tazobactam	4 to >64	16	>64	NA
	ciprofloxacin	>8 to >8	>8	>8	0
	colistin	≤0.25 to >8	0.5	1	93.2
	meropenem	8 to >64	>64	>64	0
OXA-58 (6)					
	cefiderocol	0.12–2	NA	NA	NA
	cefepime^e^	8 to >64	NA	NA	33.3
	ceftazidime/avibactam	≤0.06 to >64	NA	NA	NA
	ceftolozane/tazobactam	8 to >64	NA	NA	NA
	ciprofloxacin	>8 to >8	NA	NA	0
	colistin	≤0.25 to >8	NA	NA	66.7
	meropenem	8 to >64	NA	NA	0
GES (1)					
	cefiderocol	0.5	NA	NA	NA
	cefepime^e^	>64	NA	NA	NA
	ceftazidime/avibactam	64	NA	NA	NA
	ceftolozane/tazobactam	>64	NA	NA	NA
	ciprofloxacin	>8	NA	NA	NA
	colistin	1	NA	NA	NA
	meropenem	8	NA	NA	NA
NDM (3)					
	cefiderocol	2–8	NA	NA	NA
	cefepime^e^	>64 to >64	NA	NA	NA
	ceftazidime/avibactam	>64 to >64	NA	NA	NA
	ceftolozane/tazobactam	>64 to >64	NA	NA	NA
	ciprofloxacin	>8 to >8	NA	NA	NA
	colistin	≤0.25–2	NA	NA	NA
	meropenem	>64 to >64	NA	NA	NA

Colistin MIC measurement range was 0.25–8 mg/L. Ciprofloxacin MIC measurement range was 0.12–8 mg/L.

MIC_n_, MIC for *n*% of isolates tested; NA, not applicable (<10 isolates or no breakpoint available).

a Susceptibility data are only displayed where ≥5 isolates were available for testing and are based on EUCAST breakpoints.^5^^,^^21^

b Includes two isolates co-carrying KPC and VIM carbapenemases and eight isolates co-carrying OXA-48 and NDM carbapenemases.

c Includes one isolate co-carrying GES and VIM carbapenemases and one isolate co-carrying GES and NDM carbapenemases.

d Includes two isolates co-carrying OXA-24/40-like, OXA-58 and NDM carbapenemases and one isolate co-carrying OXA-23-like and NDM carbapenemases.

e Based on CLSI breakpoint.^17^

Against both VIM- and GES-producing *P. aeruginosa*, cefiderocol and colistin demonstrated potent activity, with 100% of isolates being susceptible to both agents. Cefiderocol and colistin were also active against non-carbapenemase-producing MDR *P. aeruginosa*, with 96.6% of isolates being cefiderocol susceptible and 100% being colistin susceptible.

Cefiderocol and colistin also demonstrated potency against *A. baumannii* producing OXA-23-like (96.3% with cefiderocol MIC ≤2 mg/L; 94.8% colistin susceptible) and OXA-24/40-like (93.2% with cefiderocol MIC ≤2 mg/L; 93.2% colistin susceptible) carbapenemases.

**Figure 3.**
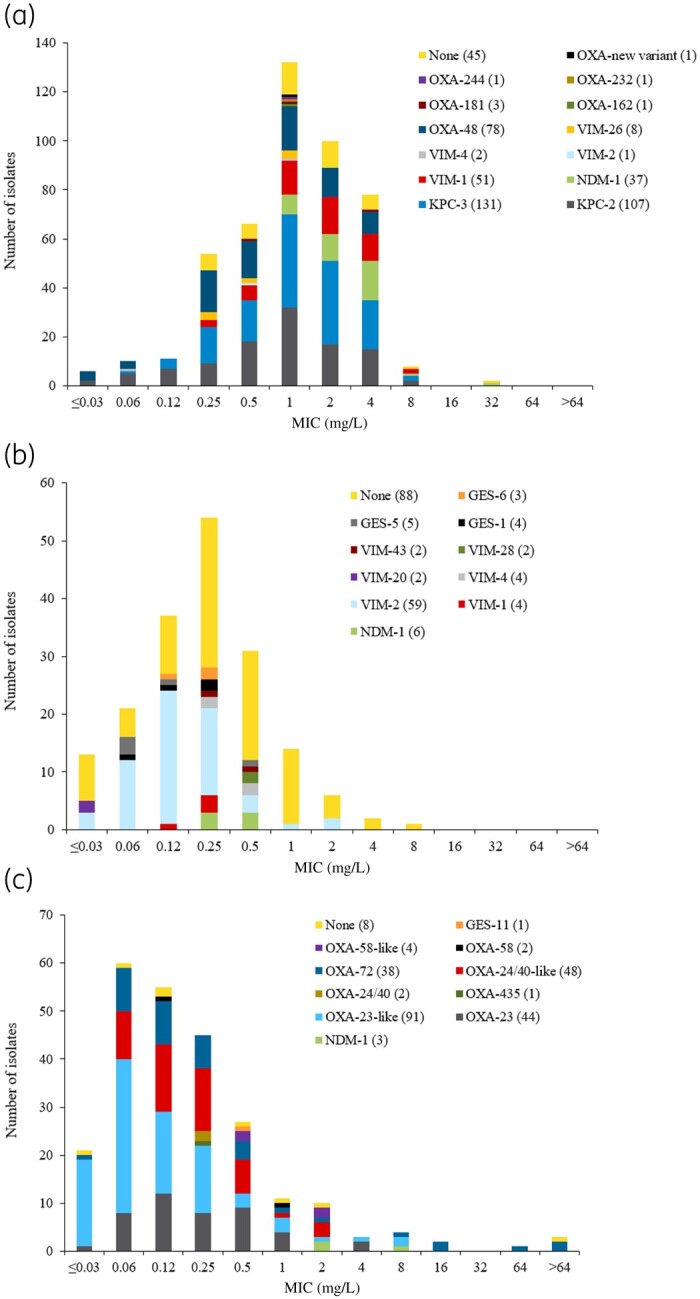
Cefiderocol MIC distribution for all carbapenemase subclasses identified in European SIDERO-CR-2014–2016 isolates, by pathogen: (a) CarbNS Enterobacterales, (b) MDR *P. aeruginosa* and (c) MDR *A. baumannii*.

The wide variety of carbapenemases produced by European Enterobacterales, *P. aeruginosa* and *A. baumannii* isolates is apparent in Figure 3. Cefiderocol MIC distributions across key carbapenemase classes are provided in Tables S2–S4.

A range of β-lactamases were identified across 99 isolates with cefiderocol MIC values of >2 mg/L, including isolates with co-carriage of multiple β-lactamases. The most common carbapenemase was KPC (*n = *39), followed by NDM (*n = *19), VIM (*n = *13), OXA-48-like (*n = *10), OXA-23-like (*n = *6) and OXA-24-like (*n = *5). Twelve isolates carried either OSBLs/ESBLs or no known β-lactamase, with eight isolates harbouring the PER-type β-lactamase.

## Discussion

For European isolates from the SIDERO-CR-2014–2016 study, cefiderocol demonstrated potent activity against a wide variety of CarbNS- and MDR-GN bacteria harbouring a range of MBLs and serine-β-lactamases.

Currently, there is an incongruity in existing cefiderocol breakpoints between EUCAST, CLSI and FDA. For example, for Enterobacterales, EUCAST: susceptible ≤2 mg/L, resistant >2 mg/L;^5^ CLSI: susceptible ≤4 mg/L, intermediate 8 mg/L, resistant ≥16 mg/L;^18^ and FDA: susceptible ≤2 mg/L, intermediate 4 mg/L, resistant ≥8 mg/L.^25^ In particular, the removal of the intermediate category by EUCAST has an impact on interpreting levels of resistance. This is illustrated by the rate of cefiderocol resistance in KPC-producing CarbNS Enterobacterales, which differs by >10-fold across the breakpoint-determining bodies: 16.4% resistant using EUCAST, 1.7% resistant using FDA and 0% resistant using CLSI breakpoints. This demonstrates that an order of magnitude difference in resistance rates can be perceived, despite the same susceptibility breakpoint being used by EUCAST and FDA, as an artefact of the lack of an intermediate breakpoint and the MIC at which the resistance breakpoint is set. This is magnified with MBLs, e.g. NDM: 48.6% (18/37) isolates are classed as cefiderocol resistant by EUCAST but only 5.4% (2/37) by FDA and 2.7% (1/37) by CLSI breakpoints. Therefore, it seems that the perceived activity of cefiderocol against KPC- and NDM-producing isolates, for example, may be affected by the differences in interpretive breakpoints for resistance.

Although there is currently some disparity between published breakpoints, pharmacokinetic/pharmacodynamic modelling predicts >90% probability of achieving 75% *fT*_>MIC_ with the recommended cefiderocol dosing regimen up to an MIC of 4 mg/L for patients with normal renal function.^26^ In Phase 3 clinical studies, the mean concentration of unbound cefiderocol exceeded 4 mg/L for the whole dosing period.

Similar isolate studies involving cefiderocol have used provisional CLSI breakpoints. In a study including 1086 CR isolates from the USA (737 KPC producers), MICs were higher for isolates of Enterobacterales with β-lactamases compared with those without, but no clear association was found between the type of β-lactamase and the MIC.^27^ Based on the CLSI provisional susceptibility breakpoint of ≤4 mg/L, 90.5% of Enterobacterales isolates were susceptible to cefiderocol. Notably, as with the European SIDERO-CR-2014–2016 isolates, the MIC_90_ for cefiderocol in Enterobacterales was 4 mg/L and the cefiderocol MIC distribution was similar to Figure 3(a). For non-fermenters, cefiderocol activity was independent of the presence of β-lactamases. A recent local study carried out in southern Spain using 231 MDR-GN isolates, including KPC, OXA, ESBL and MBL producers, found that 98% of isolates were susceptible to cefiderocol based on CLSI breakpoints.^28^ In this study, 100% of KPC-producing isolates (*n = *50) and carbapenemase-producing *K. pneumoniae* (*n = *107) were cefiderocol susceptible.

A key limitation of the SIDERO-CR study was the geographical spread and number of collection sites within countries. Isolates were selected by a limited number of sites per country based on the MDR/CR phenotype, therefore the relative frequency observed may not reflect the national prevalence. Additionally, the number of sites and isolates per country were not proportionate to population. Consequently, the isolate collection is not necessarily representative, as there may be considerable heterogeneity in the numbers and types of pathogens and mechanisms of resistance provided by different locales. However, from SIDERO-CR-2014–2016 isolate characterization, it is apparent that carbapenem resistance across Europe is associated with a diverse range of β-lactamases. Of the CR isolates included, 83.8% (729/870) produced at least one carbapenemase, with 65.2% (475/729) of carbapenemase-producing isolates also carrying ESBLs and/or OSBLs.

Current treatment options for infections caused by MDR-GN bacteria are limited. Throughout Europe, considerable heterogeneity exists across countries with respect to the types of carbapenemases contributing to CarbNS and MDR phenotypes. Even for countries where the dominant carbapenemase is KPC (e.g. Italy, Greece), recent reports have described an increasing proportion of non-KPC MBL-mediated resistance. A Rapid Risk Assessment report from the ECDC described an increase in NDM-containing carbapenemase-producing Enterobacterales in Italy,^29^ while an outbreak of XDR *K. pneumoniae* with genes encoding for OXA-48 and NDM-1 has recently been reported in the Mecklenburg-Western Pomerania state of Germany.^30^ A large outbreak of NDM-1-producing CarbNS *K. pneumoniae* was investigated across eight Greek hospitals between 2013 and 2016.^31^ The vast majority of these NDM-1-producing *K. pneumoniae* isolates were of a clonal type (ST11) similar to those identified in Bulgaria in 2015–16, indicating that the Balkan region is also at risk of increasing prevalence of MBL-producing Enterobacterales.^32^ Additionally, it has been reported that the use of ceftazidime/avibactam to treat KPC-producing *K. pneumoniae* infection has led to a shift in the carbapenemase landscape in Greece, with the incidence of MBLs increasing between 2015–17 (12.0%) and 2018 (51.1%), mainly due to VIM-producing *K. pneumoniae* becoming more prevalent.^33^ This shift from KPC- to MBL-based resistance is of concern as these strains are not susceptible to the new β-lactam/β-lactamase inhibitor combination therapies such as ceftazidime/avibactam, ceftolozane/tazobactam and meropenem/vaborbactam.^20^^,^^34–36^ These agents are known to lack efficacy against CR bacteria producing MBLs such as NDM and VIM and so lack the coverage to be used as early empirical treatment for infections suspected to involve CR-GN pathogens.^20^^,^^34–36^ Resistance to these agents was not restricted to MBL-producing isolates in the European SIDERO-CR-2014–2016 study, with 23.4% of all CarbNS Enterobacterales isolates and 65.5% of all MDR *P. aeruginosa* isolates resistant to ceftazidime/avibactam and 77.4% of all MDR *P. aeruginosa* isolates resistant to ceftolozane/tazobactam (Table 1).

Colistin, often considered a treatment of last resort, has a broad spectrum of GN activity and is frequently used for CR- and MDR-GN infections, particularly for MDR *A. baumannii.*^37^ However, colistin is undesirable for first-line empirical use due to associated renal toxicity.^38^^,^^39^ In addition, colistin-resistant strains are emerging, somewhat in *P. aeruginosa* and *A. baumannii*, but particularly in *K. pneumoniae* (including KPC, VIM, NDM and OXA producers), mediated, to some extent, by transmissible *mcr*-1 resistance.^40^ An ECDC Rapid Risk Assessment report was published in response to the increasing trend in MCR-1-mediated colistin resistance in Enterobacterales.^41^ Among SIDERO-CR-2014–2016 isolates, 123 (14.1%) were colistin non-susceptible (Figure 1), including 23.6% of Enterobacterales (28.3% of *K. pneumoniae*) and 6.4% of *A. baumannii*, with 100% of *P. aeruginosa* isolates being colistin susceptible. These results do not necessarily reflect the incidence of colistin resistance in specific geographical locations or from specific infection sources; for example, the incidence of colistin-resistant *A. baumannii* was reported to be 47.7% in a set of *A. baumannii* RTI isolates from Greece, Italy and Spain (*n = *65).^42^*In vitro* assessment of CR-GN isolates from 18 hospitals in Greece demonstrated that ∼40% of *K. pneumoniae* and *A. baumannii* isolates were resistant to colistin; 100% of these isolates had cefiderocol MICs of ≤4 mg/L.^43^ Increasing colistin resistance is a concern for clinicians as it severely restricts an already limited set of treatment options. Patients with serious CR-GN bacterial infections have a poor prognosis and require more effective, less toxic treatments than colistin, covering all MBLs and serine-β-lactamases as well as other mechanisms of resistance. Therefore, there is an urgent need for empirical treatments that cover a range of GN pathogens, coupled with rapid diagnostic techniques to identify pathogen phenotypes quickly, which will allow the prompt delivery of effective therapy.

In this study, the proportions of isolates susceptible to cefiderocol are similar to comparators in Enterobacterales and generally similar to colistin but greater than other comparators in *P. aeruginosa*. Cefiderocol retained activity against the majority of isolates harbouring MBLs, with only 3.0% (3/99) of MBL-positive CarbNS Enterobacterales isolates having MIC values >4 mg/L. The *in vitro* activity (MIC ≤2 mg/L) of cefiderocol against a range of serine-β-lactamases varied from 100% in OXA-58-producing isolates to 83.6% in KPC producers. In addition, 91.5% of isolates with no carbapenemase and 97.5% with no β-lactamase had a cefiderocol MIC ≤2 mg/L, demonstrating potent cefiderocol activity where the mechanism of resistance was not clear.

Cefiderocol demonstrated potency against the MDR *P. aeruginosa* isolates (98.3% cefiderocol susceptible), yet half carried no carbapenemase. The low prevalence of carbapenemases in SIDERO-CR-2014–2016 *P. aeruginosa* isolates aligns with recently reported data describing the prevalence of XDR *P. aeruginosa*; of 1445 *P. aeruginosa* isolates collected from 51 Spanish hospitals, 252 (17.3%) were classified as XDR and only 3.1% carried either carbapenemases or ESBLs.^44^ Resistance in non-β-lactamase-producing *P. aeruginosa* isolates in SIDERO-CR-2014–2016 is likely to be due to porin- and efflux pump-mediated mechanisms.^45^ Cefiderocol’s mechanism of entry into the bacterial cell allows it to evade these mechanisms of resistance, retaining *in vitro* activity against strains with alterations in outer membrane porins or overexpressed efflux pumps.^7^^,^^10^

In conclusion, the SIDERO-CR European dataset, coupled with the rise of resistance to existing agents and the shift towards MBL- from serine-β-lactamase-producers, demonstrates the diverse and dynamic nature of the European carbapenemase landscape. In SIDERO-CR-2014–2016, cefiderocol exhibited potent *in vitro* activity against a broad range of CarbNS and MDR pathogens. The isolates tested included β-lactamase-producing strains from all Ambler classes, with cefiderocol demonstrating activity against the key MBLs VIM and NDM, as well as against clinically important serine-β-lactamases KPC, GES and OXA, and isolates co-carrying ESBLs and OSBLs. Consequently, cefiderocol represents a key addition to the limited armamentarium available for the treatment of infections caused by CR- and MDR-GN organisms and could be a particularly valuable and timely treatment option for when resistance is apparent but the mechanism is unknown.

## Supplementary Material

dlaa060_Supplementary_Data
