# Supplementary material for: In vitro activity of the siderophore cephalosporin, cefiderocol, against molecularly characterized, carbapenem-non-susceptible Gram-negative bacteria from Europe
Source: JAC Antimicrob Resist. 2020 Aug 25;2(3):dlaa060. doi: 10.1093/jacamr/dlaa060 (PMC8210120; doi:10.1093/jacamr/dlaa060)

## Supplementary data

*Molecular characterisation of β-lactamases*

A large proportion of isolates tested as part of SIDERO-CR-2014–2016 were previously molecularly characterised for the presence of β-lactamase (*bla*) genes encoding ESBLs, plasmid-mediated AmpC-type β-lactamase, serine carbapenemases, and MBLs as described.^1,2^ As part of this study, the remaining clinical isolates that had not been previously molecularly characterised were screened for the presence of *bla* genes by PCR, followed by sequencing as described for each organism type below. For isolates for which partial β-lactamase data was already available, additional β-lactamase screening was performed to interrogate all *bla* of interest.

Enterobacterales isolates were screened for the presence of *bla* encoding ESBLs (TEM,

SHV, CTX-Ms including 5 subtypes of CTX-M-1-type, CTX-M-2-type, CTX-M-8-type,

CTX-M-9-type, and CTX-M-25-type, GES, VEB, PER including PER-1-like and PER-2-like

subtypes), plasmid-mediated AmpC-type β-lactamases (ACC, ACT, CMY, DHA, FOX, MIR,

MOX), and carbapenemases (KPC, OXA-48-like, IMP, VIM, NDM, SPM, and GIM) by

multiplex PCR using published primers^1^ and newly-designed primers that detect all

currently known PER-family genes (PERpan-F, 5’-TAGGYGTTGCMGTRTGGGG;

PERpan-R, 5’- GGTTTCRACCATCCAYTTCC). Only *bla* genes encoding the following

enzymes were amplified and sequenced in their entirety: KPC, OXA-48-like, IMP, VIM,

NDM, GES, VEB, PER, TEM, and SHV. *bla*_TEM_ and *bla*_SHV_ were screened first by limited

sequencing to identify genes encoding TEM-type and SHV-type enzymes containing amino

acid substitutions common to ESBLs at the following positions: SHV amino acid (a.a.) 146,

179, 238, 240; TEM a.a. 104, 164, 238, 240. Only *bla*_SHV_ and *bla*_TEM_ that encoded ESBLs

were sequenced.

*P. aeruginosa* isolates were screened for the presence of *bla* encoding ESBLs (TEM, SHV,

CTX-Ms including 5 subtypes of CTX-M-1-type, CTX-M-2-type, CTX-M-8-type, CTX-M-

9-type, and CTX-M-25-type, GES, VEB, PER including PER-1-like and PER-2-like

subtypes), plasmid-mediated AmpC-type β-lactamases (ACC, ACT, CMY, DHA, FOX, MIR,

and MOX) and carbapenemases (KPC, OXA-24/40-like, IMP, VIM, NDM, SPM, and GIM)

by multiplex PCR using published primers^1,2^ and the PERpan-F and PERpan-R primers

listed above. Only *bla* genes encoding the following enzymes were amplified and sequenced

in their entirety: KPC, IMP, VIM, NDM, GES, VEB, PER, TEM, and SHV. Only *bla*_SHV_ and

*bla*_TEM_ that encoded ESBLs were sequenced.

*A. baumannii* isolates were screened for the presence of *bla* encoding ESBLs (TEM, SHV,

CTX-Ms including 5 subtypes of CTX-M-1-type, CTX-M-2-type, CTX-M-8-type, CTX-M-

9-type, and CTX-M-25-type, GES, VEB, PER including PER-1-like and PER-2-like

subtypes), plasmid-mediated AmpC-type β-lactamases (ACC, ACT, CMY, DHA, FOX, MIR,

and MOX) and carbapenemases (OXA-23-like, OXA-24/40-like, OXA-48-like, OXA-58-like,

KPC, IMP, VIM, NDM, SPM, and GIM) by multiplex PCR using published primers^1,2^

and the PERpan-F and PERpan-R primers listed above. Only *bla* genes encoding the

following enzymes were amplified and sequenced in their entirety: KPC, OXA-48-like, IMP,

VIM, NDM, GES, VEB, PER, TEM, and SHV. Only *bla*_SHV_ and *bla*_TEM_ that encoded ESBLs

were sequenced.

*References*

1. Kazmierczak KM, Lob SH, Hoban DJ, *et al*. Characterization of Extended-Spectrum β-Lactamases and Antimicrobial Resistance of *Klebsiella pneumoniae* in Intra-Abdominal Infection Isolates in Latin America, 2008-2012. Results of the Study for Monitoring Antimicrobial Resistance Trends. *Diagn Microbiol Infect Dis* 2015; **82**: 209-214.

2. Nichols WW, de Jonge BL, Kazmierczak KM, *et al*. *In Vitro* Susceptibility of Global Surveillance Isolates of *Pseudomonas aeruginosa* to Ceftazidime/Avibactam (INFORM 2012 to 2014). *Antimicrob Agents Chemother* 2016; **60**: 4743-4749.

**Table S1.** European SIDERO-CR-2014–2016 bacterial isolates by source of infection

|  | **Source, *n*** | | | | | |
| --- | --- | --- | --- | --- | --- | --- |
| **Strain** | **RTI**  **(*n =* 385)** | **UTI (*n =* 157)** | **IAI (*n =* 125)** | **SSI (*n =* 89)** | **BSI (*n =* 85)** | **Other/ Unknown**  **(*n =* 29)** |
| Enterobacterales (457) | 144 | 96 | 55 | 74 | 59 | 29 |
| *Citrobacter freundii* (11) | 1 | 5 | 3 | 1 | 0 | 1 |
| *Enterobacter asburiae* (1) | 0 | 0 | 1 | 0 | 0 | 0 |
| *Enterobacter cloacae* (59) | 13 | 11 | 11 | 8 | 6 | 10 |
| *Enterobacter kobei* (4) | 0 | 0 | 2 | 1 | 0 | 1 |
| *Escherichia coli* (24) | 4 | 10 | 3 | 4 | 2 | 1 |
| *Klebsiella aerogenes* (3) | 1 | 0 | 1 | 1 | 0 | 0 |
| *Klebsiella oxytoca* (13) | 5 | 4 | 0 | 2 | 1 | 1 |
| *Klebsiella pneumoniae* (332) | 120 | 59 | 34 | 57 | 47 | 15 |
| *Serratia marcescens* (10) | 0 | 7 | 0 | 0 | 3 | 0 |
| Non-fermenters (414) | 242 | 61 | 70 | 15 | 26 | 0 |
| *Acinetobacter baumannii* (236) | 135 | 31 | 46 | 5 | 19 | 0 |
| *Pseudomonas aeruginosa* (177) | 106 | 30 | 24 | 10 | 7 | 0 |

BSI, bloodstream infection; IAI, intra-abdominal infection; MDR, multidrug-resistant; RTI, respiratory tract infection; SSI, surgical site infection; UTI, urinary tract infection.

**Table S2.** Cefiderocol MIC distribution by carbapenemase in European SIDERO-CR-2014–2016 CarbNS Enterobacterales isolates

| **Carbapenemase (*n*)** | **Number of isolates at cefiderocol MIC, mg/L** | | | | | | | | | | | | | **MIC_50_, mg/L** | **MIC_90_, mg/L** | **% with MIC ≤2 mg/L** |
| --- | --- | --- | --- | --- | --- | --- | --- | --- | --- | --- | --- | --- | --- | --- | --- | --- |
|  | **≤0.03** | **0.06** | **0.12** | **0.25** | **0.5** | **1** | **2** | **4** | **8** | **16** | **32** | **64** | **≥64** |  |  |  |
| KPC (238) | 2 | 6 | 11 | 24 | 35 | 70 | 51 | 35 | 4 | 0 | 0 | 0 | 0 | 1 | 4 | 83.6 |
| VIM (62) | 0 | 1 | 0 | 6 | 9 | 18 | 15 | 11 | 2 | 0 | 0 | 0 | 0 | 1 | 4 | 79.0 |
| NDM (37) | 0 | 0 | 0 | 0 | 0 | 8 | 11 | 16 | 1 | 0 | 1 | 0 | 0 | 2 | 4 | 51.4 |
| OXA-48-like (85) | 4 | 3 | 0 | 17 | 16 | 23 | 12 | 10 | 0 | 0 | 0 | 0 | 0 | 1 | 4 | 88.2 |
| No carbapenemase (45) | 0 | 0 | 0 | 7 | 6 | 13 | 11 | 6 | 1 | 0 | 1 | 0 | 0 | 1 | 4 | 82.2 |
| No β-lactamase (6) | 0 | 0 | 0 | 1 | 1 | 4 | 0 | 0 | 0 | 0 | 0 | 0 | 0 | NA | NA | 100 |

Includes 2 isolates co-carrying KPC and VIM carbapenemases, and 8 isolates co-carrying OXA-48 and NDM carbapenemases.

CarbNS, carbapenem-non-susceptible; ESBL, extended-spectrum β-lactamase; MIC, minimum inhibitory concentration; MIC_n_, MIC for n% of isolates tested; NA, not applicable (≤10 isolates).

**Table S3.** Cefiderocol MIC distribution by carbapenemase in European SIDERO-CR-2014–2016 MDR *P. aeruginosa* isolates

| **Carbapenemase (*n*)** | **Number of isolates at cefiderocol MIC, mg/L** | | | | | | | | | | | | | **MIC_50_, mg/L** | **MIC_90_, mg/L** | **% with MIC ≤2 mg/L** |
| --- | --- | --- | --- | --- | --- | --- | --- | --- | --- | --- | --- | --- | --- | --- | --- | --- |
|  | **≤0.03** | **0.06** | **0.12** | **0.25** | **0.5** | **1** | **2** | **4** | **8** | **16** | **32** | **64** | **≥64** |  |  |  |
| GES (12) | 0 | 4 | 3 | 4 | 1 | 0 | 0 | 0 | 0 | 0 | 0 | 0 | 0 | 0.12 | 0.25 | 100 |
| VIM (73) | 5 | 12 | 24 | 21 | 8 | 1 | 2 | 0 | 0 | 0 | 0 | 0 | 0 | 0.12 | 0.5 | 100 |
| NDM (6) | 0 | 0 | 0 | 3 | 3 | 0 | 0 | 0 | 0 | 0 | 0 | 0 | 0 | NA | NA | 100 |
| No carbapenemase (88) | 8 | 5 | 10 | 26 | 19 | 13 | 4 | 2 | 1 | 0 | 0 | 0 | 0 | 0.25 | 1 | 96.6 |
| No carbapenemase or ESBL (70) | 8 | 5 | 10 | 22 | 10 | 11 | 2 | 2 | 0 | 0 | 0 | 0 | 0 | 0.25 | 1 | 97.1 |

Includes 1 isolate co-carrying GES and VIM carbapenemases and 1 isolate co-carrying GES and NDM carbapenemases.

ESBL, extended-spectrum β-lactamase; MDR, multidrug-resistant; MIC, minimum inhibitory concentration; MIC_n_, minimum concentration inhibiting n% of isolates tested; NA, not applicable (≤10 isolates).

**Table S4.** Cefiderocol MIC distribution by carbapenemase in European SIDERO-CR-2014–2016 MDR *A. baumannii* isolates

| **Carbapenemase (*n*)** | **Number of isolates at cefiderocol MIC, mg/L** | | | | | | | | | | | | | **MIC_50_, mg/L** | **MIC_90_, mg/L** | **% with MIC ≤2 mg/L** |
| --- | --- | --- | --- | --- | --- | --- | --- | --- | --- | --- | --- | --- | --- | --- | --- | --- |
|  | **≤0.03** | **0.06** | **0.12** | **0.25** | **0.5** | **1** | **2** | **4** | **8** | **16** | **32** | **64** | **≥64** |  |  |  |
| NDM (3) | 0 | 0 | 0 | 0 | 0 | 0 | 2 | 0 | 1 | 0 | 0 | 0 | 0 | NA | NA | 66.7 |
| GES (1) | 0 | 0 | 0 | 0 | 1 | 0 | 0 | 0 | 0 | 0 | 0 | 0 | 0 | NA | NA | 100 |
| OXA-23-like (135) | 19 | 40 | 29 | 22 | 12 | 7 | 1 | 3 | 2 | 0 | 0 | 0 | 0 | 0.25 | 0.5 | 96.3 |
| OXA-24/40-like (88) | 1 | 19 | 23 | 22 | 11 | 2 | 4 | 0 | 1 | 2 | 0 | 1 | 2 | 0.25 | 2 | 93.2 |
| OXA-58 (8) | 0 | 0 | 1 | 0 | 2 | 1 | 2 | 0 | 0 | 0 | 0 | 0 | 0 | NA | NA | 100 |
| No carbapenemase (8) | 1 | 1 | 2 | 0 | 1 | 1 | 1 | 0 | 0 | 0 | 0 | 0 | 1 | NA | NA | 88.9 |
| No carbapenemase or ESBL (3) | 1 | 0 | 1 | 0 | 0 | 1 | 0 | 0 | 0 | 0 | 0 | 0 | 0 | NA | NA | 100 |

Includes 2 isolates co-carrying OXA-24/40-like, OXA-58 and NDM carbapenemases and 1 isolate co-carrying OXA-23-like and NDM carbapenemases

ESBL, extended-spectrum β-lactamase; MDR, multidrug-resistant; MIC, minimum inhibitory concentration; MIC_50_, minimum concentration inhibiting 50% of isolates tested; MIC_90_, minimum concentration inhibiting 90% of isolates tested; NA, not applicable (≤10 isolates).

**Figure S1**. Cefiderocol non-susceptible (MIC >2 mg/L) European SIDERO-CR-2014–2016 Enterobacterales and *P. aeruginosa* isolates by country.


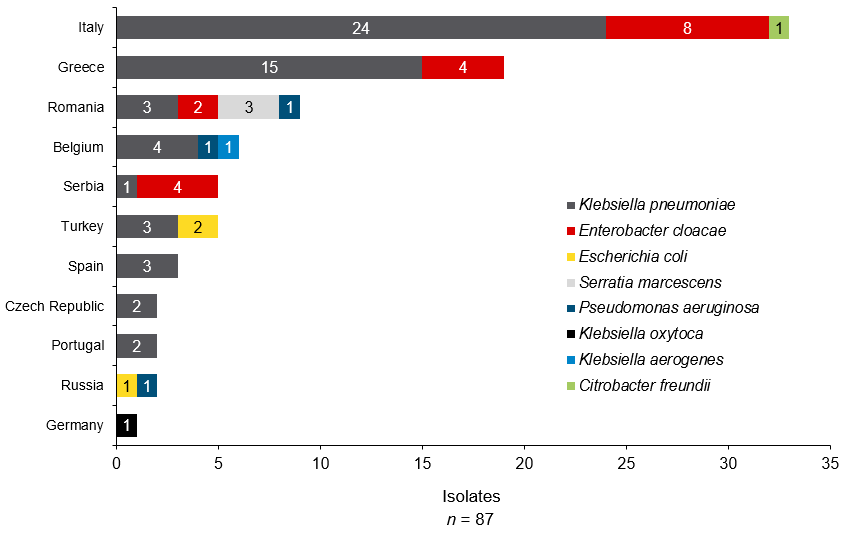


MIC, minimum inhibitory concentration.

**Figure S2.** Carbapenemase subclass production in European SIDERO-CR-2014–2016 isolates, by country, for A) KPC-producing Enterobacterales, B) OXA-48-like-producing Enterobacterales,
C) VIM-producing Enterobacterales, D) NDM-producing Enterobacterales, E) VIM-producing *P. aeruginosa*, F) OXA-23-like-producing *A. baumannii*, and G) OXA-24/40-like-producing *A. baumannii*.

A)


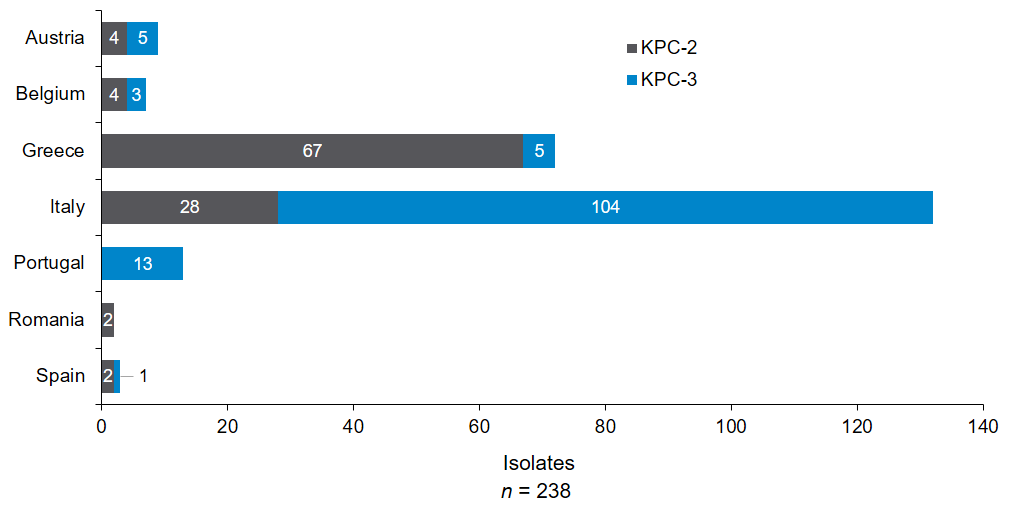


B)


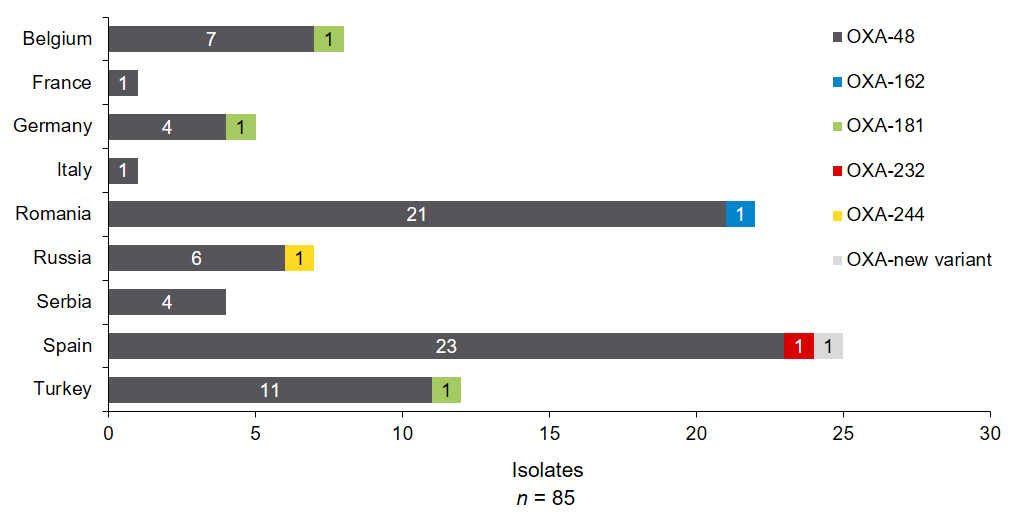


C)


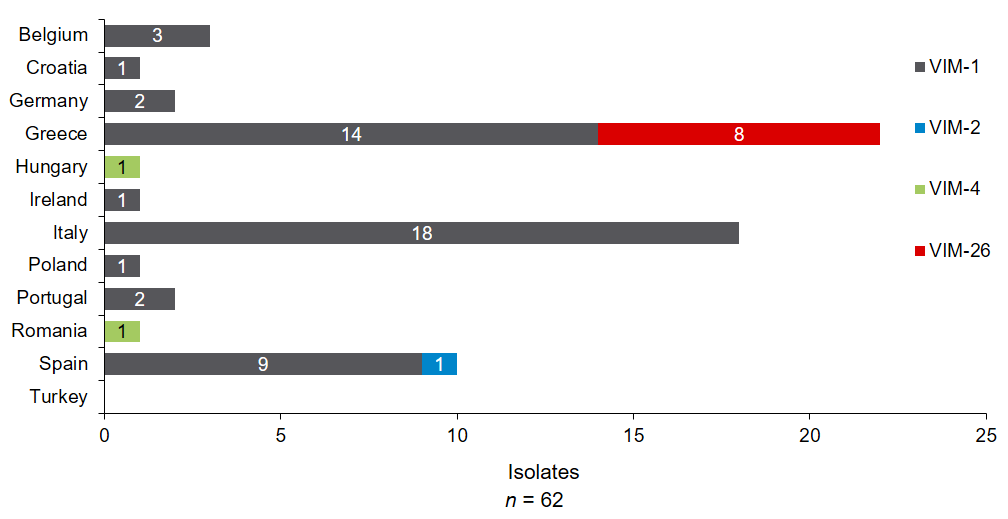


D)


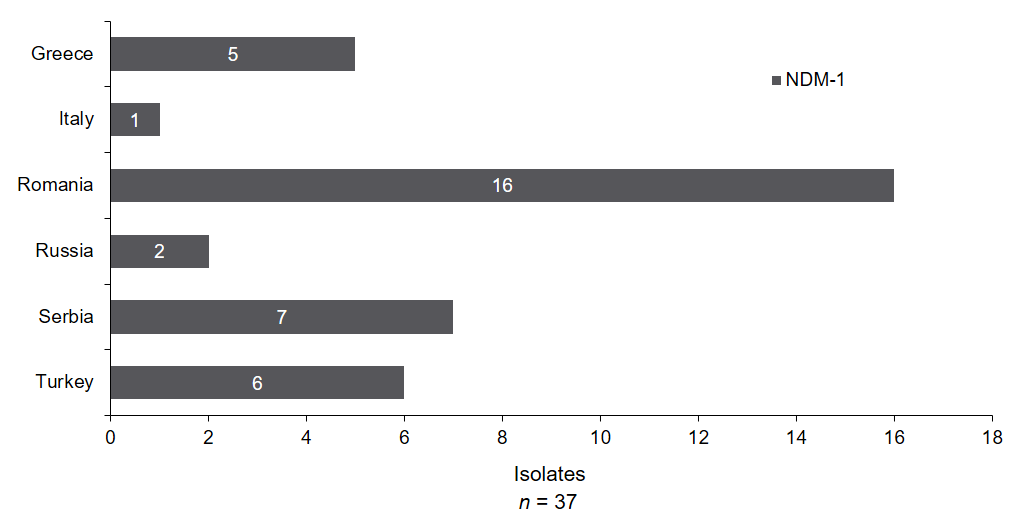


E)


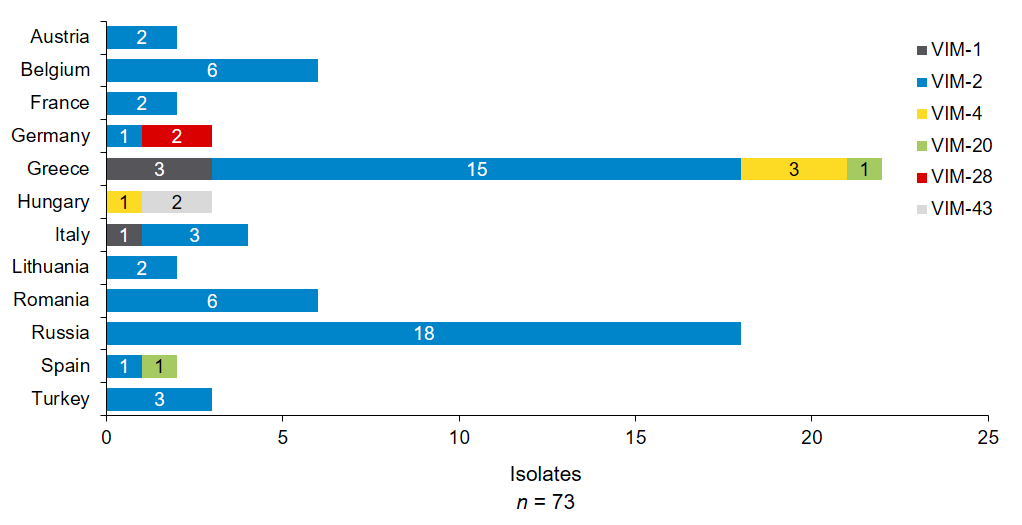


F)


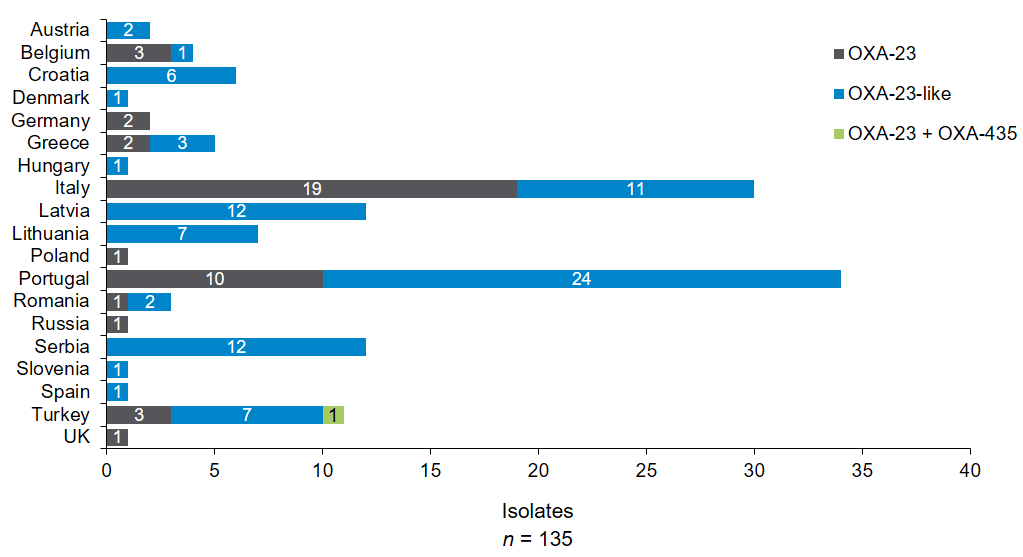


G)


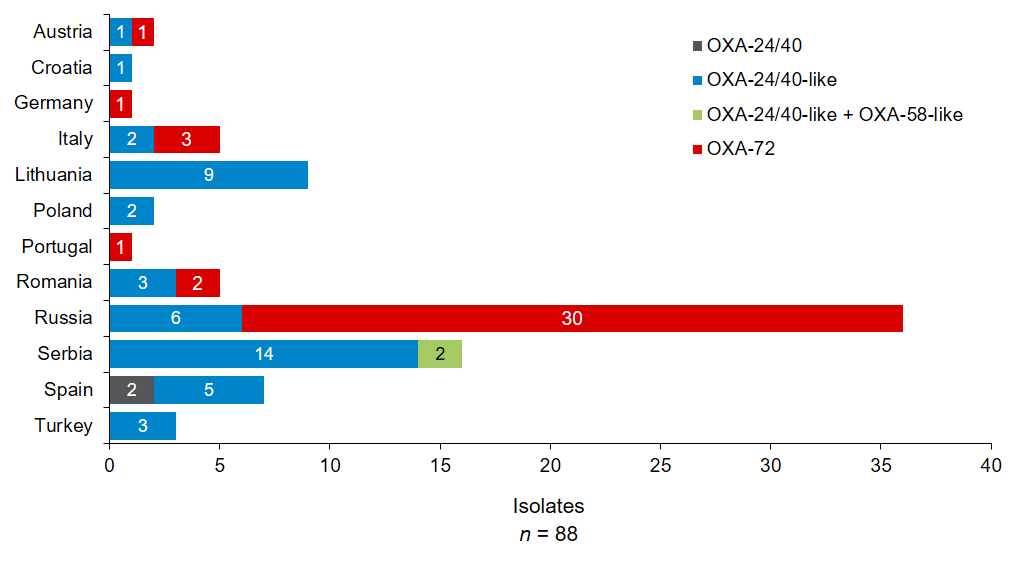

Supplement: dlaa060_Supplementary_Data [file dlaa060_supplementary_data.docx]
